# Impact of immune‐related adverse events on survival outcomes in extensive‐stage small cell lung cancer patients treated with immune checkpoint inhibitors

**DOI:** 10.1002/cam4.7188

**Published:** 2024-04-17

**Authors:** Tadashi Nishimura, Hajime Fujimoto, Takumi Fujiwara, Kentaro Ito, Atsushi Fujiwara, Hisamichi Yuda, Hidetoshi Itani, Masahiro Naito, Shuji Kodama, Kazuki Furuhashi, Akihiko Yagi, Haruko Saiki, Taro Yasuma, Tomohito Okano, Atsushi Tomaru, Motoaki Tanigawa, Masamichi Yoshida, Osamu Hataji, Hidenori Ibata, Corina N. D'Alessandro‐Gabazza, Esteban C. Gabazza, Tetsu Kobayashi

**Affiliations:** ^1^ Department of Pulmonary Medicine Mie Chuo Medical Center Tsu Japan; ^2^ Department of Pulmonary and Critical Care Medicine Mie University Faculty and Graduate School of Medicine Tsu Japan; ^3^ Department of Genomic Medicine Mie University Hospital Tsu Japan; ^4^ Respiratory Center Matsusaka Municipal Hospital Matsusaka Japan; ^5^ Department of Pulmonary Medicine Mie Prefectural General Medical Center Yokkaichi Japan; ^6^ Department of Pulmonary Medicine Kuwana City Medical Center Kuwana Japan; ^7^ Department of Respiratory Medicine Ise Red Cross Hospital Ise Japan; ^8^ Department of Immunology Mie University Faculty and Graduate School of Medicine Tsu Japan

**Keywords:** immune checkpoint inhibitor, immune‐related adverse events, small cell lung cancer

## Abstract

**Background:**

Immune checkpoint inhibitors have recently become the standard of care in the first‐line treatment of extensive‐stage small cell lung cancer. Although immune‐related adverse events have been reported to influence prognosis in non‐small cell lung cancer patients, few studies have investigated the prognostic value of immune‐related adverse events in small cell lung cancer patients. In this study, we evaluated the prognosis of patients who developed immune‐related adverse events after first‐line treatment with immune checkpoint inhibitor‐based chemotherapy for extensive‐stage small cell lung cancer.

**Methods:**

We enrolled 90 patients with extensive‐stage small cell lung cancer who received immune checkpoint inhibitor‐based chemotherapy as first‐line treatment from September 2019 to December 2022 in six hospitals in Japan. The patients were categorized into groups with and without immune‐related adverse events.

**Results:**

There were 23 patients with and 67 without immune‐related adverse events. Seventeen patients had grade 1–2 immune‐related adverse events, and nine (including overlapping cases) had grade ≥3. The most frequent immune‐related adverse event was a skin rash. The median survival time was 22 months in patients with immune‐related adverse events and 9.3 months in patients without immune‐related adverse events. The hazard ratio was 0.40 (95% confidence interval: 0.19–0.83, *p* = 0.013).

**Conclusions:**

The results of this study show that immune‐related adverse events are associated with improved survival outcomes in patients with extensive‐stage small cell lung cancer.

## Graphical abstract

Immune‐related adverse events (irAEs) after immune checkpoint inhibitors (ICI)/chemotherapy in extensive‐stage small cell lung cancer (ES‐SCLC) are linked to longer overall survival than no irAEs. irAEs indicate a significant immune response to tumor in ES‐SCLC. The prognostic impact of irAEs is independent of Eastern Cooperative Oncology Group performance status, ICI type, and regimen.

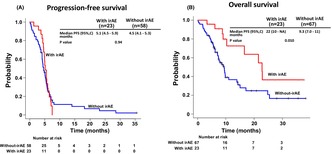

## 1 BACKGROUND

Small cell lung cancer (SCLC) is a highly aggressive type of cancer that often presents with distant metastases at diagnosis.^1^ Approximately 70% of SCLC patients are diagnosed with extensive‐stage small cell lung cancer (ES‐SCLC), which has a poor prognosis and a median survival of less than 1 year.^1^ In recent years, the introduction of immune checkpoint inhibitors (ICI) in combination with chemotherapy has improved the survival outcomes and the quality of life of ES‐SCLC patients and has become the first‐line standard of care for this disease.^2^, ^3^, ^4^, ^5^, ^6^ However, using ICI also carries the risk of immune‐related adverse events (irAE), which can affect various organs and systems.^2^, ^3^, ^4^, ^5^, ^6^ The incidence and severity of irAE vary depending on the type and regimen of ICI, tumor histology, and patient characteristics.^2^, ^3^, ^4^, ^5^, ^6^

In non‐small cell lung cancer (NSCLC), several studies have suggested that irAE is associated with a better response and longer survival in patients treated with ICI.^7^, ^8^, ^9^, ^10^, ^11^, ^12^, ^13^ However, the prognostic impact of irAE in ES‐SCLC patients who received ICI‐chemotherapy as first‐line treatment is still unclear and needs further investigation. In this study, we retrospectively analyzed the clinical data of ES‐SCLC patients who developed irAE after receiving ICI‐based chemotherapy and compared their survival outcomes with those who had no irAE.

### 1.1 Patients and methods

**FIGURE 1:**
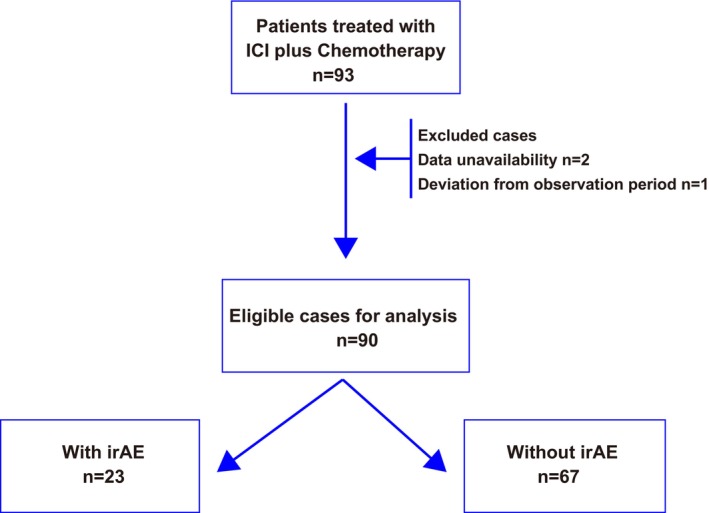
Study flow chart. ICI, immune checkpoint inhibitor; irAE, immune‐related adverse event.

This retrospective study included patients with ES‐SCLC who received first‐line therapy with ICIs plus chemotherapy between September 2019 and December 2022 at six medical centers in Japan. The chemotherapy regimen consisted of carboplatin (area under the curve: 5 mg/mL per min) and etoposide (100 mg/m^2^) for up to four cycles. The ICI doses were 1500 mg every 4 weeks for durvalumab and 1200 mg every 3 weeks for atezolizumab. Electronic medical records were used to collect patient information. Patients with insufficient information or missing observation periods were excluded from the study (Figure 1). The patients were divided into two groups based on the presence or absence of irAEs. We compared the two groups' progression‐free survival (PFS) and overall survival (OS).

### 1.2 Diagnostic workup

Patients with pathologically diagnosed small cell lung cancer and distant metastasis were defined as having ES‐SCLC. Contrast‐enhanced magnetic resonance imaging (MRI) was used to evaluate target lesions in the brain, and contrast‐enhanced computed tomography, or positron emission tomography, was used to evaluate target lesions in other organs. The Response Evaluation Criteria in Solid Tumors (RECIST) version 1.1 was applied to assess response and disease control rates. Performance status was assessed using the Eastern Cooperative Oncology Group performance status (ECOG PS). The American Society of Clinical Oncology Clinical Practice Guideline was followed to evaluate immune‐related adverse events (irAEs).^14^ Oncologists diagnosed irAEs after ruling out other possible causes based on the following criteria: (1) pathological evidence of irAEs; (2) a multidisciplinary consensus involving at least two oncologists; and (3) clinical improvement with irAE‐specific treatment.^14^, ^15^, ^16^

### 1.3 Ethics statement

Because this is a retrospective cohort study, the Institutional Review Boards of the participating hospitals approved waiving the need for informed consent by publishing an opt‐out statement on each hospital's website. The protocol for the current clinical investigation was approved by the Committee for Clinical Investigation of the following institutions: Mie Chuo Medical Center (Approval No: MCERB‐202307; Date: April 4, 2023), Mie Prefectural General Medical Center (Approval No: 2023‐S4; Date: June 7, 2023), Mie University Hospital (Approval No: H2023‐128; Date: June 21, 2023), Kuwana City Medical Center (Approval No: 232; Date: June 7, 2023), Matsusaka Municipal Hospital (Approval No: J‐242‐230526‐7‐6; Date: May 26, 2023), and Ise Red Cross Hospital (Approval No: ER2023‐72; Date: October 3, 2023).

### 1.4 Statistical analysis

Kaplan–Meier curves and log‐rank tests were used to evaluate progression‐free survival (PFS) and overall survival (OS). Landmark analyses were performed for OS survival curves for patients alive at 2, 3, and 6 months to adjust for immortal bias. The Mann–Whitney *U*‐test was used to assess continuous variables, and the Fisher exact test was used to evaluate categorical variables. The Cox proportional hazards regression model was used for univariate and multivariate analyses. A *p* value of less than 0.05 was considered statistically significant. The statistical analysis was performed using the R software package version 4.0.3 (R Development Core Team, Vienna, Austria) and the EZR version 1.54 (Saitama Medical Center, Jichi Medical University, Saitama, Japan).^17^

## 2 RESULTS

### 2.1 Patient characteristics and irAE

Ninety‐three patients were enrolled in this study, but only 90 met the eligibility criteria (Figure 1). The median age of the entire patient cohort was 72 years (range: 50–93 years). Of the total patients, 82 were male and 8 were female. Patients were stratified into two groups based on the presence or absence of immune‐related adverse events (irAEs). The irAE group comprised 23 patients, while the non‐irAE group comprised 67 (Figure 1). The baseline characteristics of patients in both groups are presented in Table 1. There were no significant differences between the two groups with regard to any of the background factors. The types and grades of irAEs are detailed in Table 2. The most frequently encountered irAEs were skin rash and hypothyroidism. Three patients experienced more than one irAE. The median time for the onset of irAEs was 75 days (range: 10–1002 days). The treatment was interrupted in three patients due to irAEs (Table 2).

**TABLE 1:** Characteristics of the patients.

Factor (%)	Group	Without irAE	With irAE	*p*‐Value
*n*		67	23	–
Age [range]		72.00 [50.00, 93.00]	73.00 [56.00, 83.00]	0.813
Sex	Male	59 (88.1)	23 (100.0)	0.108
	Female	8 (11.9)	0 (0.0)	
Smoking status	Negative	1 (1.5)	1 (4.3)	0.448
	Positive	66 (98.5)	22 (95.7)	
ECOG PS	0	20 (29.9)	11 (47.8)	0.165
	1	37 (55.2)	8 (34.8)	
	2	8 (11.9)	3 (13.0)	
	3	0 (0.0)	1 (4.3)	
	4	2 (3.0)	0 (0.0)	
First line treatment	CBDCA + etoposide + atezolizumab	42 (62.7)	10 (43.5)	0.143
	CBDCA + etoposide + durvalumab	25 (37.3)	13 (56.5)	
Total ICI cycle [range]		5.00 [1.00, 51.00]	5.00 [1.00, 37.00]	0.219
Liver metastasis	Negative	45 (67.2)	17 (73.9)	0.611
	Positive	22 (32.8)	6 (26.1)	
Malignant pleural effusion	Negative	49 (73.1)	15 (65.2)	0.595
	Positive	18 (26.9)	8 (34.8)	
Bone metastasis	Negative	48 (71.6)	14 (60.9)	0.434
	Positive	19 (28.4)	9 (39.1)	
Brain metastasis	Negative	52 (77.6)	19 (82.6)	0.771
	Positive	15 (22.4)	4 (17.4)	
Adrenal metastasis	Negative	54 (80.6)	18 (78.3)	0.771
	Positive	13 (19.4)	5 (21.7)	

Abbreviations: CBDCA, carboplatin; ECOG PS, Eastern Cooperative Oncology Group performance status; irAE, immune‐related adverse event.

**TABLE 2:** Immuno‐related adverse events.

	*n* (%)	Grade 1–2, *n* (%)	Grade ≥3, *n* (%)	Systemic steroid therapy, *n* (%)	Treatment interruption due to irAE, *n* (%)
Any	26 (100.0)	17 (100.0)	9 (100.0)	9 (100.0)	4 (%)
Rash/inflammatory dermatitis	6 (23.0)	5 (29.4)	1 (11.1)	1 (11.1)	0 (0.0)
Hypothyroidism	5 (19.2)	4 (23.5)	1 (11.1)	0 (0.0)	0 (0.0)
Pneumonitis	4 (15.3)	4 (23.5)	0 (0.0)	3 (33.3)	1 (25.0)
Hypophysitis	2 (7.6)	2 (11.7)	0 (0.0)	0 (0.0)	0 (0.0)
Polyarthritis	2 (7.6)	1 (5.8)	1 (11.1)	2 (22.2)	0 (0.0)
Hepatitis	2 (7.6)	0 (0.0)	2 (22.2)	0 (0.0)	0 (0.0)
Encephalitis	1 (3.8)	0 (0.0)	1 (11.1)	1 (11.1)	1 (25.0)
Myositis	1 (3.8)	0 (0.0)	1 (11.1)	0 (0.0)	0 (0.0)
Infusion reaction	1 (3.8)	1 (5.8)	0 (0.0)	0 (0.0)	0 (0.0)
Autoimmune hemolytic anemia	1 (3.8)	0 (0.0)	1 (11.1)	1 (11.1)	1 (25.0)
Myocarditis	1 (3.8)	0 (0.0)	1 (11.1)	1 (11.1)	1 (25.0)

### 2.2 Survival analysis

**FIGURE 2:**
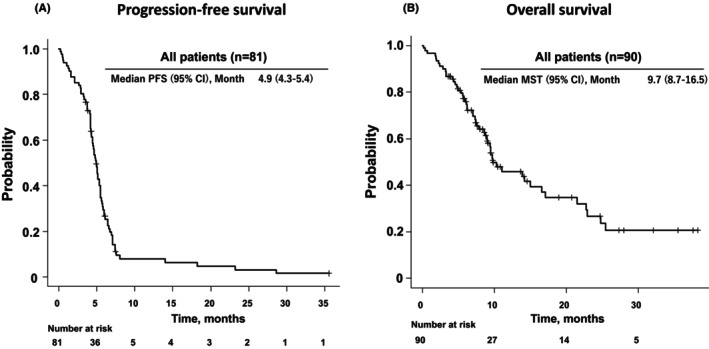
Kaplan–Meier curves of progression‐free survival and overall survival in all patients. Progression‐free survival (A) and overall survival (B) are shown. CI, confidence interval; MST, median survival time; PFS, progression‐free survival.

**FIGURE 3:**
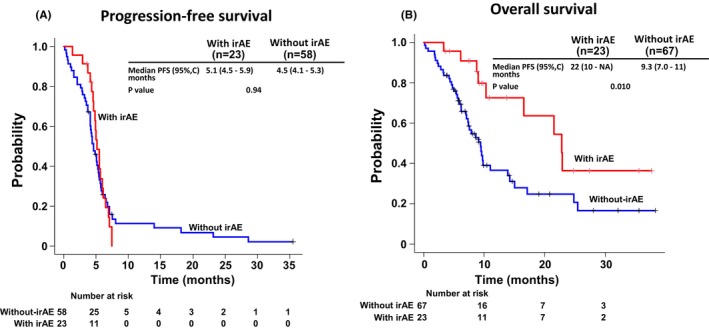
Kaplan–Meier curves of progression‐free survival and overall survival in patients with and without immune‐related adverse events. Progression‐free survival (A) and overall survival (B) are shown. CI, confidence interval; irAE, immune‐related adverse event; MST, median survival time; NA, not assessed; PFS, progression‐free survival.

**FIGURE 4:**
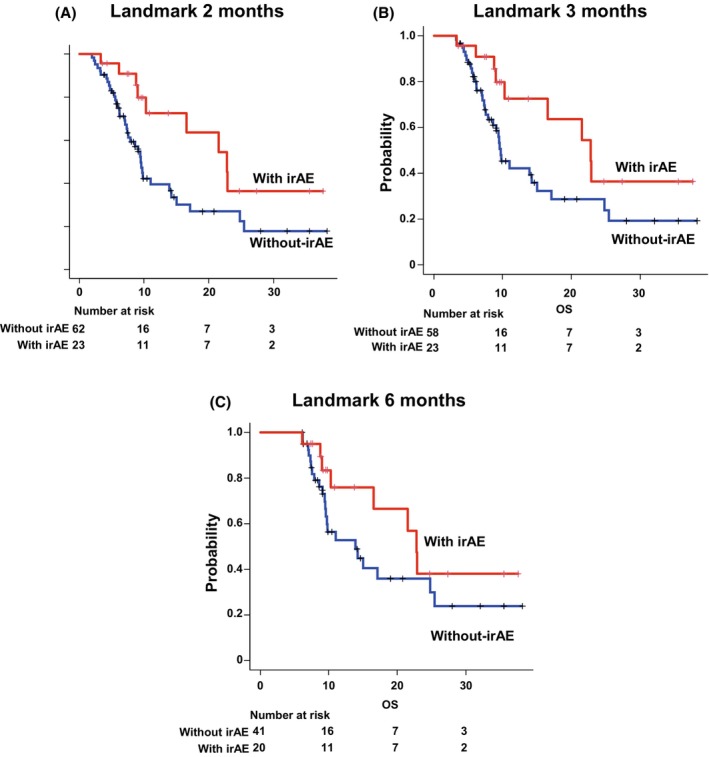
Kaplan–Meier curves for overall survival with landmark analysis. The survival curves for patients who were alive at 2 months (A), 3 months (B), and 6 months (C) after starting treatment are described. irAE, immune‐related adverse event; MST, median survival time.

In all patient groups, the PFS was 4.9 months (95% confidence interval [CI]: 4.3–5.4 months; Figure 2A), and the median OS was 9.7 months (95% CI: 8.7–16.5 months; Figure 2B). In the cohort with irAE, the median PFS was 5.1 months (95% CI: 4.5–5.9 months), whereas in the group without irAE, it was 4.5 months (95% CI: 4.1–5.3 months; *p* = 0.941; Figure 3A). The median OS was significantly extended in the irAE group, with a median survival time of 22.8 months (95% CI: 10.3‐not assessed months) compared to the group without irAE, which had a median OS of 9.3 months (95% CI: 7.0–11.0 months; *p* = 0.010; Figure 3B). It is noteworthy that the number at risk in the group without irAE at the initiation of PFS and OS is different. This discrepancy can be attributed to patients who did not undergo CT scans at the conclusion of treatment, rendering them ineligible for Response Evaluation Criteria in Solid Tumors (RECIST) evaluation. Consequently, the data for PFS was incomplete. However, as the date and time of death were meticulously documented, the count of events for OS is higher. This incongruity emanates from the inclusion of real‐world cases in the study. A landmark analysis was conducted to account for immortal time bias. In the 2‐ and 3‐month landmark analyses, significant extensions in OS were observed in the irAE group compared to the group without irAE (Figure 4A,B; Table 3). However, the 6‐month landmark analysis did not reveal any statistically significant differences (Figure 4C; Table 3).

**TABLE 3:** Landmark analysis for overall survival.

	With irAE		Without irAE		*p* Value (Log‐rank)	Hazard ratio (95% CI)
	*n*	MST (95% CI), months	*n*	MST (95% CI), month		
Landmark 2 months	23	22.8 (10.3‐NA)	62	9.4 (7.3–14.2)	0.023	0.43 (0.21–0.91), *p* = 0.027
Landmark 3 months	23	22.8 (10.3‐NA)	58	9.6 (7.8–14.9)	0.045	0.47 (0.22–1.0), *p* = 0.050
Landmark 6 months	20	22.8 (10.3‐NA)	41	13.9 (9.4–24.8)	0.16	0.56 (0.25–1.2), *p* = 0.17

Abbreviations: CI, confidence interval; MST, median survival time.

**FIGURE 5:**
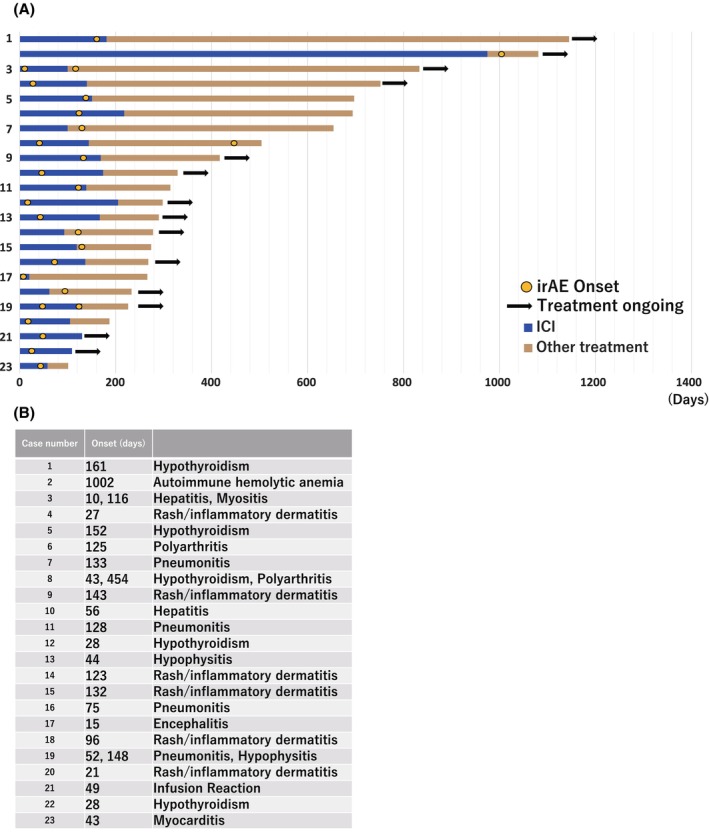
Swimmer plots depicting patients with immune‐related adverse events. Individual swimmer's plot for patients with immune‐related adverse events (irAE; A) and details of the irAE (B). ICI, immune checkpoint inhibitor.

Survival curves were also analyzed based on the grade of irAEs and prednisolone treatment. However, no significant findings were identified, probably due to the limited number of patients (Figure S1). Swimmer's plots are described in Figure 5A,B. All but two cases completed immune checkpoint inhibitor (ICI) treatment. All but one case experienced irAE within 160 days.

### 2.3 Univariate and multivariate analyses

Univariate and multivariate analyses were performed using the Cox proportional hazards regression. Both univariate and multivariate analyses revealed that Eastern Cooperative Oncology Group performance status (ECOG PS) of 2 or higher and the absence of irAEs were poor prognostic factors for OS (Table 4).

**TABLE 4:** Univariate and multivariate analyses.

Factor	Overall survival			
	Univariate analysis		Multivariate analysis	
	Hazard ratio	*p* Value	Hazard ratio	*p* Value
Age				
<70	Reference	0.14	Reference	0.23
≥70	1.57 (0.86–2.87)		1.48 (0.78–2.81)	
Sex				
Male	Reference	0.75	Reference	0.72
Female	1.16 (0.46–2.93)		0.84 (0.32–2.20)	
ECOG performance status				
0–1	Reference	0.0037	Reference	0.00059
≥2	2.71 (1.38–5.32)		4.04 (1.82–8.96)	
Brain metastasis				
Negative	Reference	0.56	Reference	0.19
Positive	0.81 (0.41–1.62)		0.58 (0.26–1.32)	
Liver metastasis				
Negative	Reference	0.17	Reference	0.69
Positive	1.55 (0.83–2.87)		1.18 (0.53–2.64)	
Malignant pleural effusion				
Negative	Reference	0.8	Reference	0.83
Positive	1.08 (0.6–1.94)		1.08 (0.52–2.23)	
Bone metastasis				
Negative	Reference	0.59	Reference	0.34
Positive	0.84 (0.45–1.58)		0.71 (0.34–1.45)	
Adrenal metastasis				
Negative	Reference	0.21	Reference	0.13
Positive	1.50 (0.8–2.83)		1.69 (0.85–3.37)	
irAE				
Without	Reference	0.013	Reference	0.00082
With	0.40 (0.19–0.83)		0.24 (0.10–0.55)	

Abbreviations: ECOG PS, Eastern Cooperative Oncology Group performance status; irAE, immune‐related adverse event.

## 3 DISCUSSION

This study provides novel and important insights into the prognostic role of irAEs in patients with ES‐SCLC who received first‐line treatment with ICIs plus chemotherapy. Previous studies have shown that irAEs are associated with improved survival outcomes in patients with non‐SCLC and in patients with SCLC who received second‐line ICI therapy.^7^, ^8^, ^9^, ^10^, ^11^, ^12^, ^13^, ^14^ However, this is the first study to demonstrate that irAEs are also a favorable prognostic factor for patients with ES‐SCLC who received first‐line ICI plus chemotherapy, suggesting that irAEs may be a surrogate marker of treatment efficacy and a predictor of long‐term survival benefit for this aggressive disease.

There have been numerous reports exploring the correlation between irAEs and prognosis.^7^, ^8^, ^9^, ^10^, ^11^, ^12^, ^13^, ^14^ Shimozaki et al. found that patients with irAE in solid tumors, including non‐small cell lung cancer, malignant melanoma, renal cell carcinoma, and gastric cancer, exhibited superior progression‐free survival (PFS) and overall survival (OS).^11^ Another study on non‐small cell lung cancer demonstrated excellent PFS and OS in patients with irAE, particularly those treated with nivolumab.^8^, ^9^, ^10^, ^13^ Recently, Socinski et al. conducted a pooled analysis of patients enrolled in a clinical trial using atezolizumab, analyzing 2503 patients with a landmark analysis to avoid bias.^12^ Their findings indicated a prognostic impact of irAE, showing a hazard ratio of 0.69 for OS in patients with irAE compared to those without irAE.^12^ The underlying mechanisms of how irAEs influence prognosis are still unclear. One possible explanation is that ICIs activate exhausted T cells to cross‐react with both tumor antigens and self‐antigens, resulting in irAEs and enhanced anti‐tumor immunity.^18^, ^19^ Therefore, patients with irAEs may have more tumor cell death and antigen activity induced by ICIs, which could lead to durable tumor control and prolonged survival. Another possible explanation is that genomic variants associated with irAEs may modulate both the immune response and the tumor biology, affecting both irAEs and prognosis.^20^ Further studies are needed to elucidate the molecular and immunological mechanisms of irAEs and their impact on survival outcomes.

Our study also has implications for the optimal use of ICIs in ES‐SCLC treatment. The IMPOWER133 trial showed that adding ICIs to carboplatin and etoposide improved survival in patients with ES‐SCLC compared to chemotherapy alone.^21^ However, other trials failed to show any benefit of ICIs as a second‐line treatment for ES‐SCLC.^22^, ^23^, ^24^, ^25^ Thus, it seems that the efficacy of ICIs is mainly observed during the first‐line treatment and that subsequent ICI therapy may not be effective for patients who did not respond to first‐line ICI plus chemotherapy. Therefore, it is crucial to identify biomarkers that can predict the response to first‐line ICI plus chemotherapy and guide the selection of patients who may benefit from this treatment. We propose that irAEs may be a biomarker, as they reflect the immune activation and the tumor sensitivity to ICIs. We also believe that genomic variants associated with irAEs may be another potential biomarker, as they may influence both the immune response and the tumor biology.

Our study has some limitations that should be acknowledged. First, this is a retrospective study that may be subject to selection bias and confounding factors. Second, the number of patients with irAEs is small, which limits the statistical power and generalizability of our findings. Third, some irAEs may occur long after the administration of ICIs, which may lead to a selection bias in favor of patients who survive longer after treatment.^26^ We performed a landmark analysis to adjust for this bias and found a trend toward a better prognosis in the irAE group, but the long‐term follow‐up may not be accurate due to the small sample size. Fourth, we could not perform multivariate or subgroup analyses to account for other factors that may influence the prognosis, such as the number and type of irAEs, the use of steroids, and the line of therapy.^10^, ^11^, ^14^ These factors may have different effects on the survival outcomes of patients with irAEs.

In our report, the incidence of Grade 3 or higher adverse events exceeded that observed in clinical trials. Fujimoto et al. conducted a prospective observational study utilizing real‐world data of ES‐SCLC with ICI to chemotherapy in Japan.^27^ Their findings indicated that patients not eligible for inclusion in clinical trials experienced a higher frequency of Grade 3 or higher adverse events.^27^ Therefore, we attribute the higher occurrence of Grade 3 or higher adverse events in our data to the inclusion of real‐world patients, encompassing demographics not typically represented in clinical trials, such as elderly patients and those with poor performance status.

In summary, the present study unveils for the first time that patients afflicted with ES‐SCLC who experienced irAEs following ICI as a first‐line treatment exhibited significantly improved OS compared to patients without irAEs. This observation underscores the potential of irAEs as a surrogate marker for treatment effectiveness and as a pivotal prognostic factor in this challenging disease. Nevertheless, it is imperative to acknowledge the limitations of our study, including its small sample size, retrospective design, and the absence of genomic analysis. Consequently, our findings warrant validation through larger cohorts, prospective study designs, and comprehensive genomic profiling of irAE‐related variants.

## AUTHOR CONTRIBUTIONS

**Tadashi Nishimura:** Conceptualization (equal); resources (equal); writing – original draft (equal). **Hajime Fujimoto:** Conceptualization (equal); supervision (equal). **Takumi Fujiwara:** Conceptualization (equal); supervision (equal). **Kentaro Ito:** Data curation (equal); resources (equal); supervision (equal). **Atsushi Fujiwara:** Data curation (equal); validation (equal). **Hisamichi Yuda:** Data curation (equal); validation (equal). **Hidetoshi Itani:** Data curation (equal); resources (equal). **Masahiro Naito:** Data curation (equal); resources (equal); validation (equal). **Shuji Kodama:** Data curation (equal); resources (equal). **Kazuki Furuhashi:** Data curation (equal). **Akihiko Yagi:** Data curation (equal). **Haruko Saiki:** Data curation (equal). **Taro Yasuma:** Data curation (equal). **Tomohito Okano:** Data curation (equal). **Atsushi Tomaru:** Data curation (equal); resources (equal). **Motoaki Tanigawa:** Data curation (equal); resources (equal). **Masamichi Yoshida:** Data curation (equal); resources (equal). **Osamu Hataji:** Data curation (equal); resources (equal). **Hidenori Ibata:** Conceptualization (equal); resources (equal); supervision (equal). **Corina N. D'Alessandro‐Gabazza:** Data curation (equal); writing – review and editing (equal). **Esteban C. Gabazza:** Conceptualization (equal); writing – review and editing (equal). **Tetsu Kobayashi:** Conceptualization (equal); data curation (equal); supervision (equal).

## CONFLICT OF INTEREST STATEMENT

The authors declare no conflicts of interest related to the present work. However, some of the authors have conflicts of interest outside the scope of the present study. K. Ito received lecture fees from Eli Lilly, Boehringer Ingelheim, Takeda Pharmaceutical, Chugai Pharmaceutical, AstraZeneca, Pfizer, Merk Sharp & Dohme (MSD), Ono Pharmaceutical, and Taiho Pharmaceutical. O. Hataji received grants from AbbVie, AstraZeneca, Boehringer Ingelheim, Byer, Chugai Pharmaceutical, Eli Lilly, Fukuda Denshi, GlaxoSmithKline, Insmed, Janssen Pharmaceutical, Kyorin Pharmaceutical, Merk Sharp & Dohme (MSD), Novartis, Ono Pharmaceutical, Sanofi, and Takeda Pharmaceutical, and lecture fees from AstraZeneca, Boehringer Ingelheim, Daiichi Sankyo, Fukuda Denshi, GlaxoSmithKline, Kyorin Pharmaceutical, Merck Biopharma, Merk Sharp & Dohme (MSD), Meiji Seika Pharma, Mitsubishi Tanabe Pharma, Nippon Kayaku, Nippon Chemiphar, Novartis, Ono Pharmaceutical, Sanofi, Takeda Pharmaceutical, Taiho Pharmaceutical, and Boehringer Ingelheim. E.C. Gabazza received funding from the 2022 and 2023 Takeda Foundations. T. Kobayashi received grants from Chugai Pharma and lecture fees from AstraZeneca.

## Supporting information


Figure S1.

## Data Availability

All data are available upon reasonable request to the first author of the article.
