# Supplementary material for: Impact of immune‐related adverse events on survival outcomes in extensive‐stage small cell lung cancer patients treated with immune checkpoint inhibitors
Source: Cancer Med. 2024 Apr 17;13(8):e7188. doi: 10.1002/cam4.7188 (PMC11022147; doi:10.1002/cam4.7188)
Supplement: Supplementary file 1 — Figure S1. [file CAM4-13-e7188-s001.docx]

**Impact of Immune-Related Adverse Events on Survival Outcomes in Extensive-Stage Small Cell Lung Cancer Patients Treated with Immune Checkpoint Inhibitors**

Tadashi Nishimura, Hajime Fujimoto, Takumi Fujiwara, Kentaro Ito, Atsushi Fujiwara, Hisamichi Yuda, Hidetoshi Itani, Masahiro Naito, Shuji Kodama, Kazuki Furuhashi, Akihiko Yagi, Haruko Saiki, Taro Yasuma, Tomohito Okano, Atsushi Tomaru, Motoaki Tanigawa, Masamichi Yoshida, Osamu Hataji, Hidenori Ibata, Corina N D'Alessandro-Gabazza, Esteban C Gabazza, Tetsu Kobayashi.


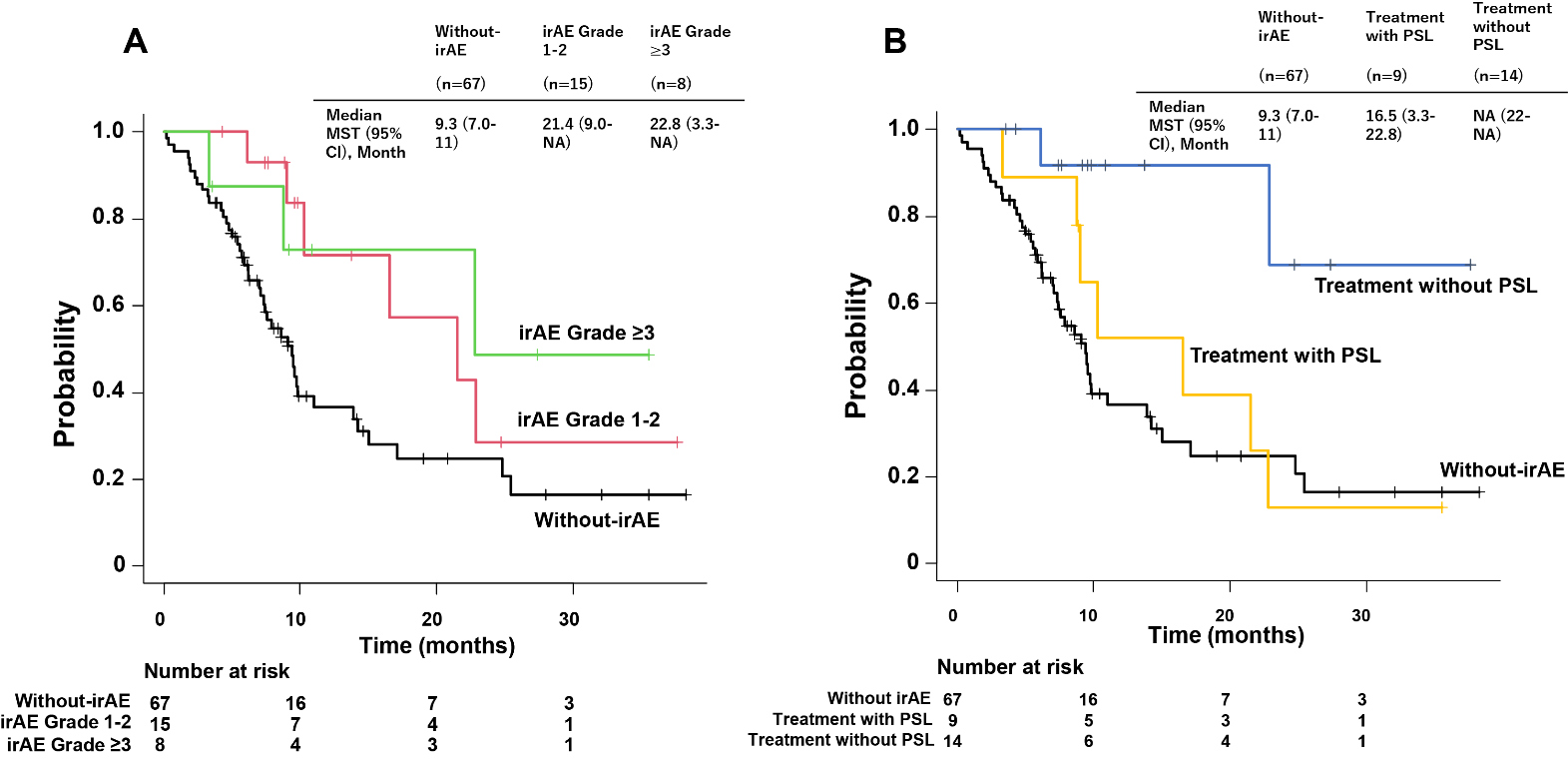


**Supplementary Figure 1. Survival curves based on the grade of irAEs and prednisolone treatment.** Kaplan-Meier curves for overall survival of patients based on irAE grade (A) or treatment with prednisolone (B). irAE: immune related adverse event, NA: not assessed, PSL: prednisolone.
